# Numerical Prediction of the Effect of Thermal Plume of a Standing Human on the Airborne Aerosol Flow in a Room: Assessment of the Social Distancing Rule

**DOI:** 10.1007/s41810-022-00165-2

**Published:** 2022-11-11

**Authors:** Mamdud Hossain, Nkemjika Chinenye-Kanu, Nadimul H. Faisal, Anil Prathuru, Taimoor Asim, Snehashish Banik

**Affiliations:** 1 School of Engineering, Robert Gordon University, Garthdee Road, Aberdeen, AB10 7GJ UK; 2 Aberdeen Royal Infirmary, Foresterhill, Aberdeen, AB25 2ZN UK; 3 School of Medicine, Medical Sciences and Nutrition, University of Aberdeen, Aberdeen, AB24 3FX UK

**Keywords:** SARs-COV-2, Airborne aerosol flow, Thermal plume, Room

## Abstract

The purpose of the study is to investigate the dispersion of droplet nuclei/aerosol which are produced during coughing and continuous talking to quantify the risk of infection due to airborne disease transmission. A three-dimensional modelling of aerosol transport due to human respiratory activities such as coughing and talking within a room environment has been simulated using CFD technique. An inert scalar transport equation was used to represent aerosol cloud, while turbulence was modelled with the $k-\epsilon$ turbulence model. A modified Wells–Riley equation was used to calculate the risk of infection based on quanta emission concept. The spatial and temporal distribution of aerosol cloud within the room is initially driven by the upward flowing thermal plume surrounding the human, but later driven by the flow field constrained by the walls and cooler air movement. While the cough generated aerosols are concentrated in a smaller space within the room, the continuous talk generated aerosols are distributed throughout the room. Within an indoor environment, 2 m distancing will not be enough to protect healthy people from aerosols coming from an infected person due to continuous talking with prolonged exposure.

## Introduction

As the world tries to get back to normal working during current coronavirus (COVID-19) pandemic in the transmission of severe acute respiratory syndrome coronavirus 2 (SARS-CoV-2) virus, all forms of restrictions including socialising indoors are being lifted. However, the virus has shown continued mutation leading to multiple waves of the on-going pandemic. The potential for indoor transmission of the new highly transmissible variants of SARS-CoV-2 by pre-symptomatic and asymptomatic individuals could potentially increase and trigger series of waves of the pandemic especially among unvaccinated and immune compromised vaccinated individuals. In all such transmission, the modes of transmission depend on the sizes of the virus laden droplets/aerosol released through the nostrils and mouths of infected individuals and the physical processes that they become exposed to once they are in the environment.

Mittal et al. (2020) worked on formation of exhaled droplets and their drying and evaporation process. On the generation of the droplets, the emphasis was on two mechanisms. The first mechanism is the instabilities of the mucus lining namely surface-tension-driven Rayleigh–Plateau instability, shear-driven Kelvin–Helmholtz instability, acceleration-driven Rayleigh–Taylor instability and Rayleigh–Taylor instability. The second mechanism is the droplet formation due to breakup of the fluid lining during the opening of closed respiratory passage namely the terminal bronchioles, possibly the larynx and then the mouth where the movement and contact of the tongue and lips imitates the breakup mechanism for instance during violent sneezing. Although these internal fluid dynamics mechanisms are not discussed in detail in the present study, it is worthwhile mentioning them since they contribute to the number density, velocity, size distribution, viral load concentration of the eventually exhaled droplets and consequently the dispersion of the droplets in the environment.

The particles sizes and the associated thermal and flow dynamics that they are subjected to has been examined in different investigations and much more recently to arrive at an accurate definition of the characteristics of the airborne transmission of COVID-19. Mittal et al. (2020) identified large droplets size to be > 100 µm and small droplets to be < 100 µm based on their review of previous findings in literature. They also reported that the critical size that separate the large and small droplets group exist in a range of 50–150 µm due to prevailing temperature and humidity variations. Depending on the exhalation velocity, the large droplets fall ballistically on to surfaces which are about 3–6 feet from the source. The range of large droplets expired during sneezing may however, travel beyond 20 feet due to high exhalation velocity (Xie et al. 2007; Bourouiba et al. 2014) before settling on surfaces as well mainly due to gravity. Mostly, the large droplets will sediment before drying thereby contaminating the surfaces with left-over residues after complete drying. Vuorinen et al. (2020) described such droplets to be larger than 200 μm in size. The medium sized and small droplets which are caught up in the turbulent cloud due to respiratory jet are suspended in the cloud longer than the large droplets and so they travel further than the large droplets (Hossain and Faisal 2021). The implication of being suspended in air is that the originally small droplets and the droplet nuclei created by the evaporation of the medium sized droplets before they had the opportunity to settle on a lower surface, would further float upwards due to buoyancy. Several publications also (Mittal et al. 2020; Asadi et al. 2019, 2020; Stadnytskyi et al. 2020) also showed that these droplets that are initially small or larger droplets that dry up rapidly outside the mouth to form light/small droplets nuclei are able to linger in the air similar to aerosols or other sufficiently small particles.

The rate of evaporation of droplets depends on the temperature difference between the droplet surface and ambient temperature and the humidity of the environment. For instance, since a solid particle of *d* ≤ 50 µm released at a height of 1.6 m in still air will sediment in 30 s and a droplet of similar size completely evaporates in 3 s forming water vapour at relative humidity of 50%, hence a respiratory mucus droplet of same size will also completely evaporate before sedimentation. Therefore, a droplet nucleus capable of being transported in air current due to buoyancy and potentially consisting of virions and solid residue remains at the end of the rapid evaporation (Vuorinen et al. 2020). Ferretti et al. (2020) reported that transmission from individuals who are pre-symptomatic, symptomatic, and asymptomatic are 46%, 32% and 10% respectively while transmission from the environment was about 6%. However, asymptomatic, and environmental transmission were lacking confirmation. Such transmissions refer to droplets that are initially small and released from the non-exerting activities of asymptomatic individuals or larger droplets that dry almost immediately outside the mouth into light/small droplet nuclei in the environment. The droplet nuclei linger in the air like aerosols or other sufficiently small particles (Asadi et al. 2019, 2020; Stadnytskyi et al. 2020). Although airborne transmission might not be a principal transmission mode, understanding it and quantifying its effect is needed to develop a holistic epidemiology of COVID-19.

Studies have combined the analyses of droplet/aerosol release rate, computational fluid dynamics, and the science of virus survivability to determine the time dependent airborne transmissibility of COVID-19. Such studies have attempted to accurately define the exact concentration of the infectious components in expiratory particles/aerosols by describing a quantity called ‘quanta’ per unit time or unit volume. Buonanno et al. (2020) described viral load as quanta emission rate. The quantum is defined as “the dose of airborne droplet nuclei required to cause infection in 63% of susceptible persons”. To obtain a high-fidelity account of the transient quanta concentration in an indoor space, Qian et al. (2009) and Buonanno et al. (2020) rightly considered the space ventilation rate, particle deposition/settling rate on the floor/surfaces and the viral inactivation/death rate. These factors defined the infectious virus removal rate from the ambient air.

The small droplets and droplet nuclei which result from the evaporation process are in the size range 1–10 µm which enables it to be transported in ambient air current and remain suspended for hours in indoor environment such as classrooms, homes, offices, elevators, malls, mass transport vehicles such as aircrafts and buses (Mittal et al. 2020). In a typical residential indoor location with minimal ventilation, the air flow in the human breathing zone which refers to the region in the last few centimetres (boundary layer) closest to the human body becomes critical for the effective airborne transmission of COVID-19 (Sun et al. 2021). The temperature gradient between the human body and the ambient air creates an upward flow within the microenvironment. This constantly rising airflow is known as the human thermal plume and has been proven to control the dispersion and transport of aerosols in the breathing zone (Sun et al. 2021). Hence, in a calm indoor environment with little or no ventilation, the contribution of the thermal plume to the overall airflow is expected to become very important since the main driver of the air current will be buoyancy due to natural convection. Therefore, the assessment of the effect of indoor air current and natural convection contributed by human thermal plume is crucial in prediction of indoor airborne transmission of COVID-19 (Mittal et al. 2020).

Dbouk and Drikakis (2021) have investigated droplets dispersion inside a lift from a mild cough of a person using a Eulerian–Lagrangian particle tracking method, while the turbulence was modelled with standard $k-\epsilon$ model. The droplets size distribution from the cough was modelled using a Rosin–Rammler distribution. Main finding from the simulations indicate that the droplets dispersion depends on the locations of inlet and outlet of the ventilation vents and the location of the individual. The presence of an air purifier does not eliminate the dispersion of droplets. Overall conclusion from the study is that the placement and design of air purifier and ventilators significantly affect the droplets dispersion and should be carefully designed to minimise dispersion. Mirikar et al. (2021) applied RNG $k-\epsilon$ turbulence model and Eulerian–Lagrangian particle tracking model to investigate the droplets transport in a typical office meeting/conference room occupied by three people, with one of them infected. The simulations show that most of the droplets fall on the table, released due to coughing of the infected person. Komperda et al. (2021) investigated droplets transport inside a large dental clinic due to wall mounted jet wall ventilation under aerosols generating scaling procedure using a Eulerian–Lagrangian modelling techniques. They have used $k-\epsilon$ model for turbulence prediction. Their simulation results show that droplets larger than 60 µm fall on the ground, but smaller droplets/aerosols have large residence time of 7.31 min and travel 24.45 m contaminating the whole dental clinic. Nazari et al. (2021) have investigated dispersion of sneeze released droplets within an underground car parking. A Eulerian–Lagrangian CFD model has been used with a fixed size of droplet. The use of jet fan disperses the droplets within the car park, the safe zone resides near the edge of the parking space where fresh air ducts are located. Zhang et al. (2021) investigated the transport of aerosols with an urban bus. They have modelled the aerosols transport as an inert scalar. They have found the use of HVAC in the bus are significant contributors to aerosols dispersion within the bus.

Burgmann and Janoske (2021) have investigated aerosols transport inside the classrooms. They gathered data of artificial aerosols dispersion within the classroom and used that data to validate CFD simulations. In the CFD simulations, they have used SST $k-\omega$ model for handling turbulence and an inert scalar particle to simulate aerosols transport. Their simulation results show that the air purifier system leads to a significant reduction of airborne particles in the room depending on the location of the infected person. Talaat et al. (2021) investigated the aerosols transport inside the cabin of an airplane using CFD model with RNG $k-\epsilon$ model for turbulence and Lagrangian particle tracking for aerosols transport. Simulation results show that using sneeze shields with full capacity can reduce the aerosol transmission to a level below that of the reduced capacity without sneeze shields. Vourinen et al. (2020) have implemented a Large Eddy Simulation (LES) model and an inert scalar transport model for treating turbulence and aerosols transport respectively for simulating aerosols transport inside a supermarket aisle and have identified the domain of elevated risks can extended up to 4 m. Sarhan et al. (2021) has investigated 1 µm size droplet transport within a room for breathing and speaking and concluded that 1.5 m is not adequate to prevent getting infected. Their modelling approach is based on $k-\epsilon$ turbulence model with Lagrangian particle tracking for turbulence and droplet transport respectively.

From above, it can be assessed that indoor spaces such as air cabins, buses, restaurants, classrooms, etc. have highly complex flows dominated by large recirculation zones. This is caused by ventilation system as well as thermally driven follow effects due to humans. These flows significantly affect the distribution of aerosols. In the present study, the transport of aerosols released due to coughing and talking inside a living room has been investigated. The simulations consider the convective flow generated by human body and its effects on aerosols dispersion. Human body generated thermal plume effects have often been neglected in most of the studies. Furthermore, both temporal and spatial distribution of aerosols have been studied leading to identification of domain of elevated risk using Wells–Riley equation.

## Methodology

The Reynolds-Averaged Navier–Stokes (RANS) equations have been numerically solved to predict the turbulent flow fields inside the room, where the turbulence has been treated by standard $k-\epsilon$ model. The distribution of temperature within the room has been calculated using an energy equation, while the distribution of aerosols has been predicted using a scalar transport equation. Two different approaches have been used by researchers for exhaled droplets and aerosols transport. A Lagrangian particle tracking with droplet size distribution with droplets evaporation has been used by Dbouk and Drikakis (2021), Mirikar et al. (2021). They are primarily focussed on determining large droplets fall-off within a shorter time interval. On the other hand, Vuorinen et al. (2020) and Zhang et al. (2021) used a Eulerian framework, solving a transport equation of a passive scalar to represent aerosol concentration per unit volume to investigate aerosol dispersion over a longer distance and time scale. Since the focus of the present study is on aerosols dispersion within a room over a longer timescale, the later approach has been adopted. However, it should be noted that the larger droplets will not follow the movement of airflows perfectly. Those would drop-off from the initial airflow and drop to the ground quickly without contributing to airborne transmission.

### Governing Equations of Fluid Flows

The turbulence airflow field has been predicted by solving conservation of mass and momentum equations:

1 $$\nabla \cdot {\varvec{u}}=0$$

2 $$\frac{\partial {\varvec{u}}}{\partial t}+\nabla \cdot \left(uu\right)=-\nabla p-{\varvec{g}}\cdot {\varvec{x}}\nabla \left(\frac{\rho }{{\rho }_{o}}\right)+\nabla \cdot \left[{\nu }_{\mathrm{eff}}(\nabla {\varvec{u}}+\nabla {{\varvec{u}}}^{T}\right]$$

where, ${\varvec{u}}$ is the velocity vector, $p$ is the pressure, ${\varvec{g}}$ the acceleration due to gravity, $\rho$ and ${\rho }_{o}$ are the local and nominal density respectively. ${\nu }_{\mathrm{eff}}$ is the kinematic viscosity that accounts for both laminar and turbulent viscosities.

The local density is calculated from the local temperature according to $\frac{\rho }{{\rho }_{o}}=1-\beta \left(T-{T}_{o}\right)$. Here, $\beta$ is the thermal expansion coefficient and for air its value is $\beta =3\times {10}^{-3}$ K^−1^. $T$ and ${T}_{o}$ are the local and nominal temperatures, respectively. The local temperature is determined by solving a temperature equation as:

3 $$\frac{\partial T}{\partial t}+\nabla \cdot \left({\varvec{u}}T\right)=\nabla \cdot ({\alpha }_{eff}\nabla T)$$

where, ${\alpha }_{eff}=\frac{{\nu }_{t}}{{Pr}_{t}}+\frac{\nu }{Pr}$. ${Pr}_{t}=0.9$ and $Pr=0.71$ represent the turbulent and laminar Prandtl numbers respectively. The $k-\epsilon$ turbulence model has been used for determining the turbulent viscosity (Launder and Spalding 1974).

When a human breathes, talks, coughs, sneezes, a large number of droplets are released into surrounding atmosphere. Depending on the exhalation activities, the number and sizes of droplets vary, however, under normal conditions of breathing and talking or even coughing most of the exhaled droplet sizes are less than 1 µm and seldom larger than 5 µm (Papineni and Rosenthal 1997; Fabian et al. 2008). While larger droplets (> 100 µm) fall on the ground due to gravity while simultaneously evaporating and reducing in sizes, the smaller droplets (< 10 µm) travel passively with the carrier fluids. The focus of the current work is on the transport of micron sized droplets (aerosols) due to buoyancy driven flow generated by human body temperature. The aerosols are considered as a passive scalar and their transport is governed by a convection–diffusion equation as,

4 $$\frac{\partial C}{\partial t}+\nabla \cdot \left(uC\right)=\nabla \cdot \left({D}_{\mathrm{eff}}\nabla C\right)$$

where, $C$ is the aerosols concentration per unit volume, ${D}_{\mathrm{eff}}=\frac{{\nu }_{t}}{S{c}_{t}}+\frac{\nu }{Sc}$. $S{c}_{t}=Sc=1$ are the turbulent and laminar Schmidt numbers respectively. A Schmidt number of unity indicates that the aerosols diffuse at the same rate as the momentum and this is valid for droplets sizes between 1 and 10 µm.

The equations governing the fluid flow, heat transfer and aerosol concentration are solved using Ansys Fluent version 20. A second order upwind scheme has been used for discretising convection terms, while time dependent terms have been solved using an implicit scheme with a time step of 10^–4^ s. The SIMPLE algorithm has been used to couple velocity and pressure discretised equations.

### The Infection Risk Model

The prediction of risk of airborne transmission of Covid-19 has been performed using a modified Riley et al. (1978) and adopted from Buonanno et al. (2020). In this concept, the viral load emitted is expressed in terms of quanta emission rate, where a quantum is defined as the dose of airborne droplet nuclei required to cause infection in 63% of susceptible person. Considering the rate of change of quanta levels within a control volume, the governing differential equation can be solved to evaluate quanta concentration in an indoor environment at the time $t$ as:

5 $$n\left(t\right)=\frac{E{R}_{q}\cdot I}{IVRR\cdot  V}+\left({n}_{o}-\frac{E{R}_{q}\cdot I}{IVRR}\right)\cdot \frac{{e}^{-\left(IVRR\cdot  t\right)} }{V}$$

where, ${n}_{o}$ represents the initial number of quanta in the space, $I$ is the number of infectious subjects, $V$ is the volume of the indoor environment considered and $E{R}_{q}$ is the quanta emission rate (quanta per h), which is a characteristic of the specific disease/virus under investigation. $IVRR$ represents the infectious virus removal rate within the indoor space. The $IVRR$ is made of three contributions (1) air exchange rate via ventilation (2) particle deposition surfaces (due gravitational setting) and (3) the viral inactivation rate. In the present study, the room did not have artificial ventilation and the particle deposition through CFD modelling and thus, only the viral inactivation rate has been considered explicitly. Based on the COVID-19 virus half-life of 1.1 h, the virus inactivation rate has been determined as 0.63 h^−1^ (Buonanno et al. 2020). The quanta emission rate is dependent on the type of virus, the virus load on the mouth and expiratory activities of breathing, talking, coughing and sneezing and physical activities of resting, standing, exercise. Buonanno et al. (2020) has given an estimate for COVID-19 quanta emission rate (quantum/h) for a standing person with talking to have a quanta emission rate of 237 (quantum/h). To determine the infection risk ($R \%)$ as a function of the exposure time $t$ of susceptible people, the quanta concentration can be integrated over time according to Wells–Riley equation:

6 $$R=1-{e}^{-IR{\int }_{0}^{T}n\left(t\right)\mathrm{d}t}$$

where, $IR$ is the inhalation rate of the exposed subject which depends on the activity level. $T$ is the total time of exposure. This formulation assumes that exhaled aerosols are instantaneously and evenly distributed within the room. However, CFD modelling allows to determine the aerosols temporal and spatial evolution within the room and thus Eq. (6) can be modified to determine spatial distribution of risk within an enclosed space by,

7 $$R=1-{e}^{-IR\cdot V{\int }_{0}^{T}n\left(t\right)C(t)\mathrm{d}t}$$

where, $V$ is the volume of the room and $C(t)$ is the aerosols concentration distributions.

### Computational Setup

**Fig. 1:**
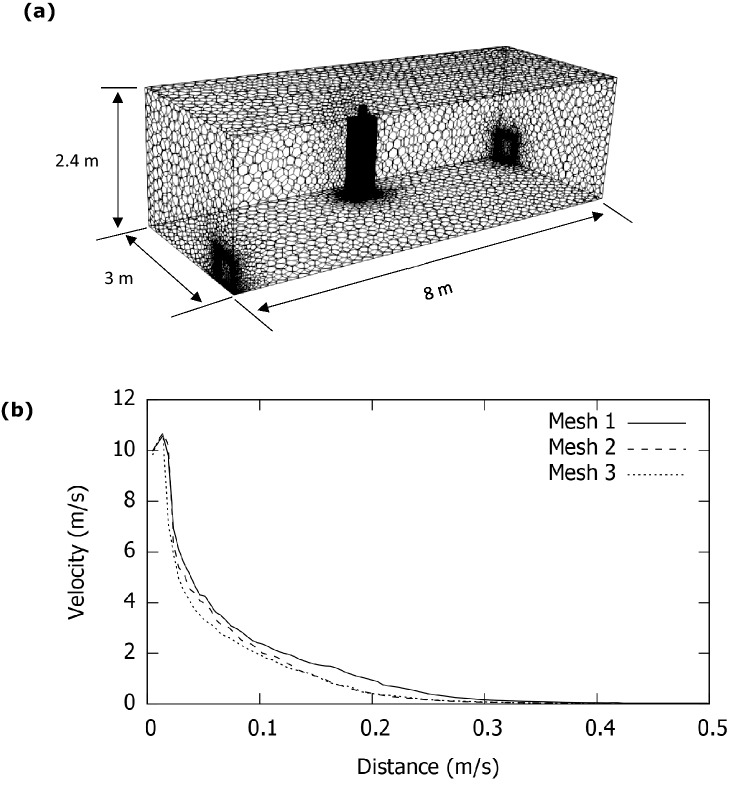
**a** Geometry and mesh showing a person standing inside a room, and **b** grid independency test using velocity profile

A typical size of a standard living room in the UK has been simulated with room dimension of 8 m × 3 m × 2.4 m. A standing human has been represented by a 1.72 m tall manikin within the room. The manikin has been modelled as a cuboid body with the head and neck modelled as a cylinder. The mouth of the manikin has been modelled as a rectangle slot with a size of 0.04 m × 0.00484 m according to Dbouk and Drikakis (2020a). Figure 1 shows the geometry, the human location and the generated mesh. Two exhalation conditions of coughing and breathing have been simulated in the present study. The exhalation velocity of 10 m/s over a period of 0.3 s has been set for the flow exhalation from the manikin mouth according to Dbouk and Drikakis (2020a) and Xie et al. (2007), while for talking a continuous velocity of 5 m/s was specified at the inlet according to Xie et al. (2007) and Asadi et al. (2019). In the simulation, the human body temperature was 36 °C, wall and room temperature were 18^O^C and the mouth released airflow temperature was 39 °C. The temperature difference between human body and the room gives a Grashof number of 2.88 × 10^12^ indicating a turbulent flow.

Three different meshes were created to study the effects of mesh distribution in the results. The meshes were polyhedral with hexahedral meshes near the human body to capture boundary layer. The number of cells or control volumes in the mesh were: 491,094 (mesh 3), 797,380 (mesh 2) and 1,684,971 (mesh 1). Figure 1b shows steady state axial velocity of jet due to an inlet velocity of 10 m/s (cough). The successive refinement of mesh reduces the differences is velocity predictions. In the present study, 1,684,971 cells have been used in further simulations. For a similar room, Sarhan et al. (2021) used 1,125,000 cells to achieve mesh independent solution.

## Results and discussion

Two exhalation conditions of coughing and breathing have been simulated in the present study. The focus of the present study is to understand how the body generated thermal plume influences the aerosol transport, and thus a thorough understanding of the air circulation and temperature distribution within the room is needed.

**Fig. 2:**
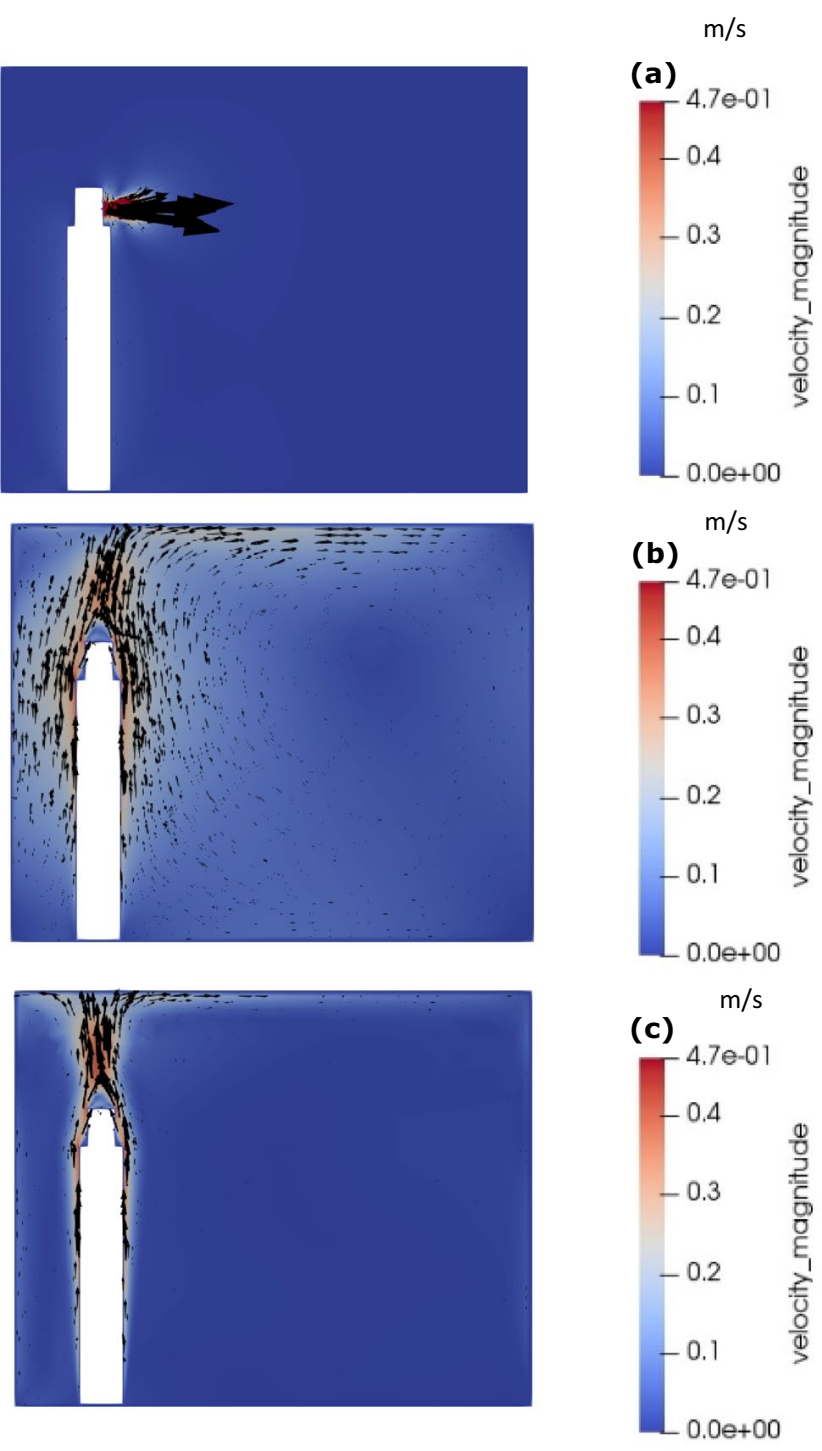
Velocity contours and vectors for coughing: **a** 0.3 s, **b** 25 s, and **c** 5 min

Figure 2 shows the velocity magnitude and vector plots for coughing condition. The vector arrows were not scaled and thus it shows the direction of velocity. At 0.3 s, the cough ceases with the flow coming out of the mouth at 10 m/s. Outside the momentum jet, the flow is driven by buoyancy showing an upward jet with two recirculation zones, above and below the cough jet due to roll-up of the shear layers at the periphery of the jet. At 25 s, the flow is predominantly driven by buoyancy with an upward motion along the body and a big recirculation zone is observed within the room. The velocity magnitude within this recirculation zone is observed to be higher than at 0.3 s due to dissipation of cough jet into the ambient air. At 5 min, the effect of cough diminishes and the recirculation zones that were observed at 25 s breaks down into more complex flow patterns.

**Fig. 3:**
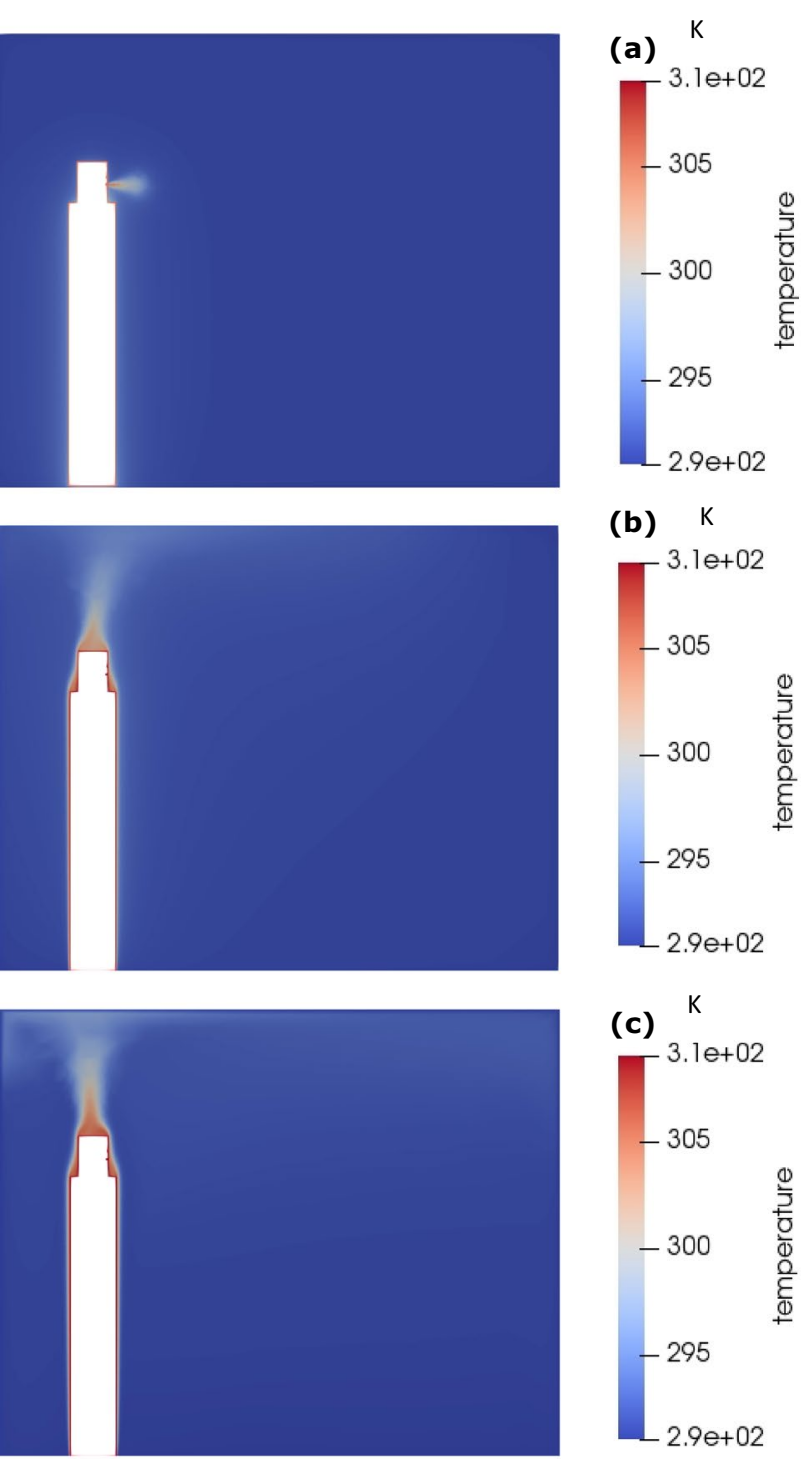
Temperature distribution inside the room for coughing: **a** 0.3 s, **b** 25 s, and **c** 5 min

Figure 3 shows the temperature distribution within the room due to coughing. At 0.3 s, the hot air is released into the room due to the cough, however, the hot air then dissipates within the room, as discussed above. The thermal gradient observed in the room is in the direction of the cough, as expected. At 25 s a thermal plume is observed around the body due to natural convection heat transfer established between the body and the surrounding and it is further consolidated at 5 min. The thermal plum is responsible for driving the flow upward and then creating recirculating motion within the room, and thus, the initial thermal gradient in the direction of the cough changes direction, upwards-to-downwards, which is expected in case of flows driven by natural convection.

**Fig. 4:**
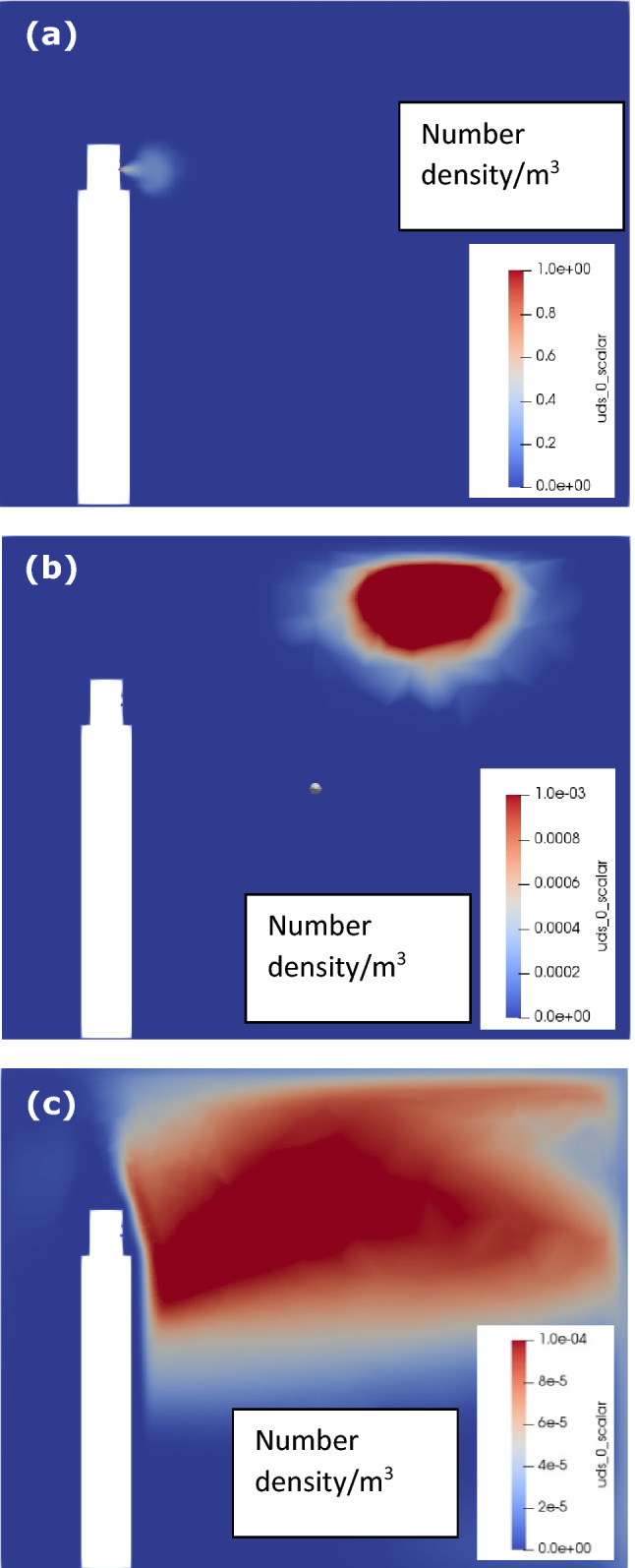
Concentration distribution within the room for coughing: **a** 0.3 s, **b** 25 s, and **c** 5 min

Figure 4 shows cough generated aerosol distribution within the room. At 0.3 s, a mushroom cloud like aerosol structure is observed attached to the mouth with the peak aerosol concentration of 1 at the core. The aerosol cloud is carried upward and towards the corner after 25 s due to the combined effect of natural convection and coughing momentum. At 5 min, excessive dissipation of momentum and diffusion of aerosols within the room leads to non-uniformly distributed aerosols, while still under the effect of natural convection due to their higher temperature, and thus, are seen to be present in the upper half of the room. At this instance, the concentration reduces significantly to 10^–4^.

**Fig. 5:**
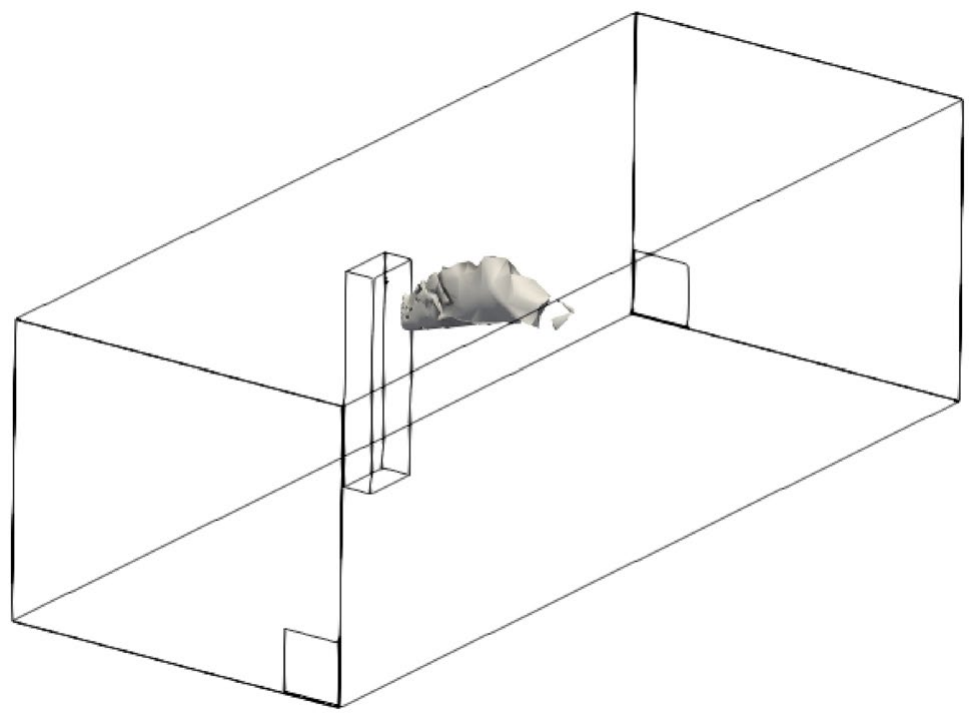
3D contours of concentration for coughing after 5 min

Figure 5 shows the 3D contour of aerosol concentration of 10^–4^. The figure clearly shows that the aerosol diffuses quickly entraining air within the cloud due to flow recirculation, occupying a small volume within the room. The rise of the cloud is clearly visible (due to natural convection).

**Fig. 6:**
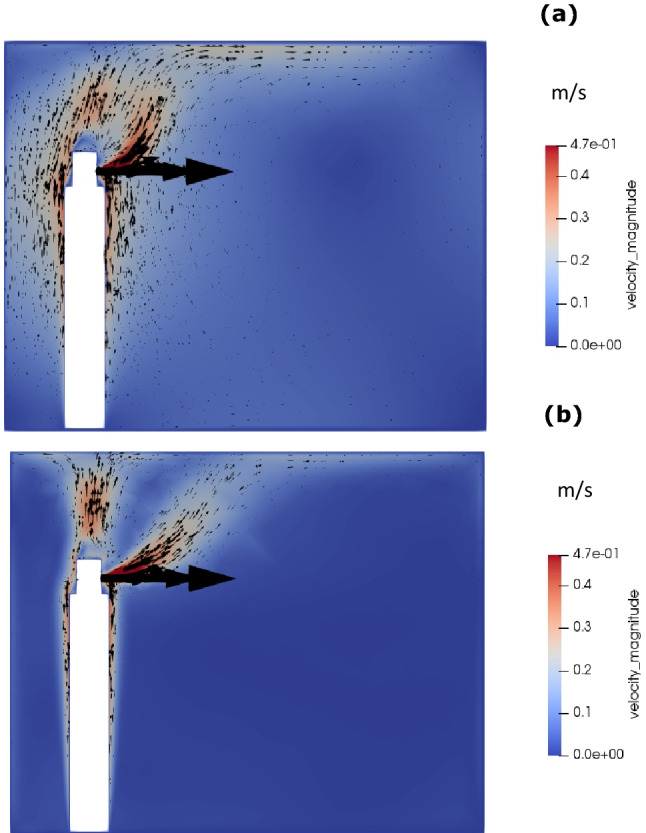
Velocity contour and vectors for continuous talking: **a** 25 s, and **b** 5 min

**Fig. 7:**
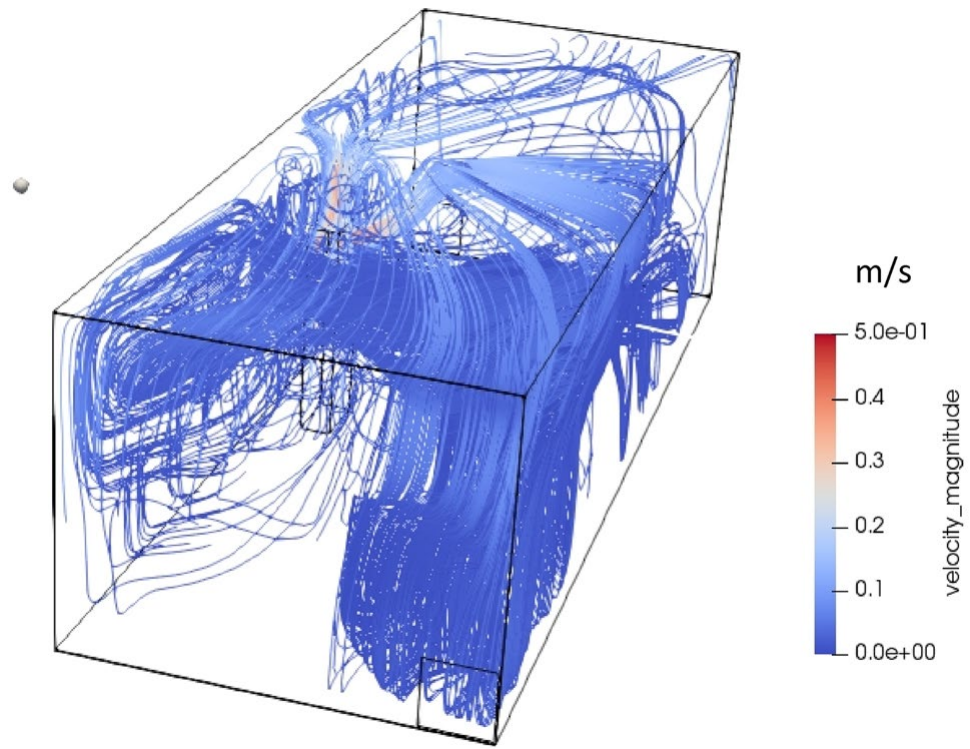
The airflow streamlines velocities at 5 min for continuous talking

Figure 6 shows the velocity contour and vectors due to continuous talking of 5 min. The continuous talking has been described by a continuous velocity, without considering the transient flows created by pauses. This figure shows that there are two streams of flows, one from the mouth and the other generated by thermal plume along the body moving upward. The two streams merge near the roof, creating a complex recirculatory flow. Though at 5 min, it seems there is no flow at the lower half of the room, however, the actual flow features are not captured on this 2-dimensional plane. At lower half of the room, the airflows from the left to right of the room and at top half, air flows from right to left of the room. As shown in Fig. 7, the velocity streamlines clearly shows that the airflow inside the room is dominated by body generated thermal plume and speaking generated flows. Further, the natural convection takes the flow upwards towards the roof and spread through the room. It is noteworthy that the wall-effects are significant contributors in the generation of these recirculating zones, leading to complex three-dimensional flow interactions and subsequent propagation and dissipation. Naturally buoyant air creates a state air circulation within the room that has a significant contribution to the aerodynamics of aerosols as explained below.

**Fig. 8:**
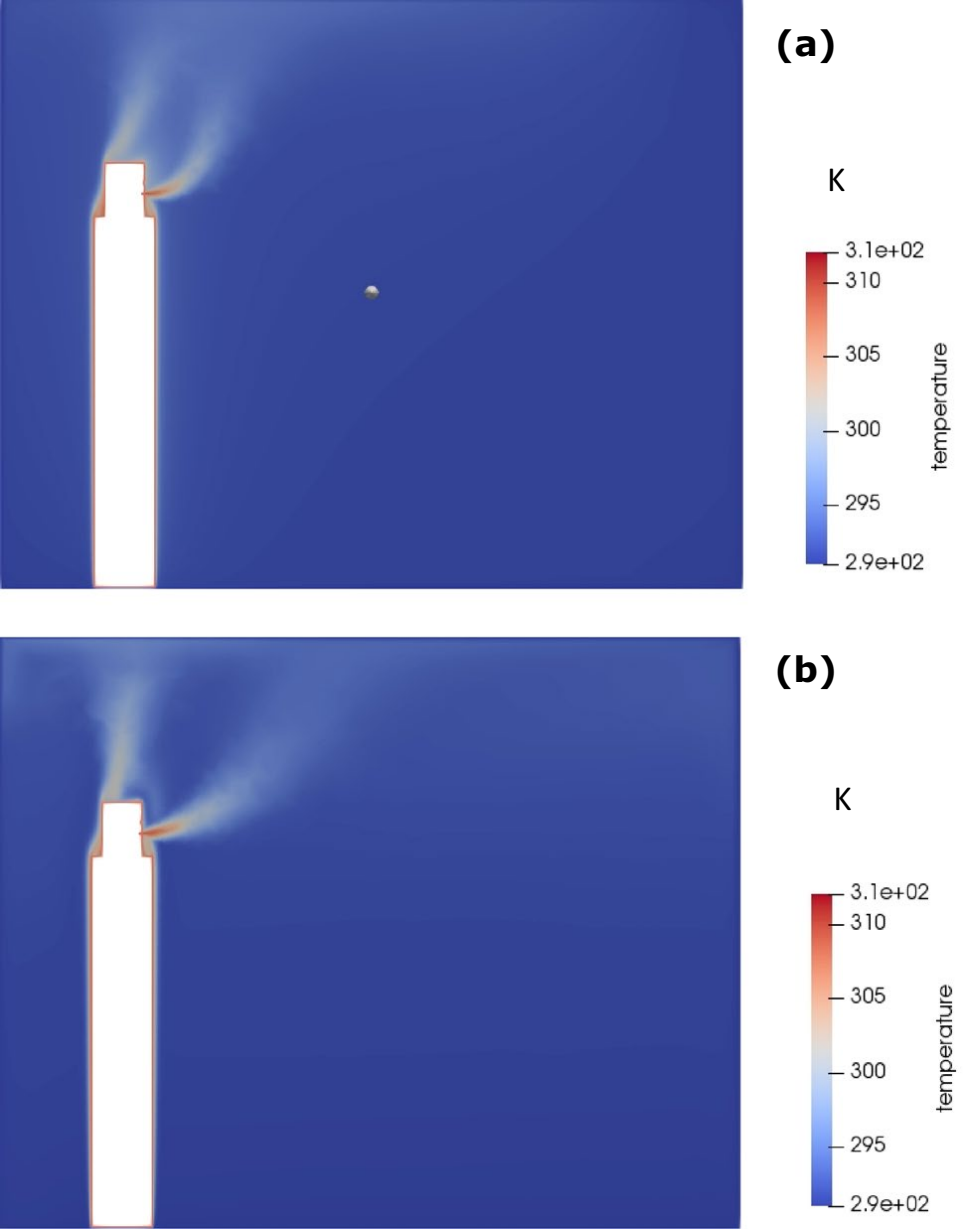
Temperature contour for continuous talking: **a** 25 s, and **b** 5 min

Figure 8 shows the temperature profiles within the room. The figure shows that there are two streams of heat source within the room, one is body generated thermal plume and the other is mouth generated upward hot air jet. Due to continuous talking, both these streams continue to co-exist, while maintaining the thermal gradient in the upward-downward direction, as discussed above.

**Fig. 9:**
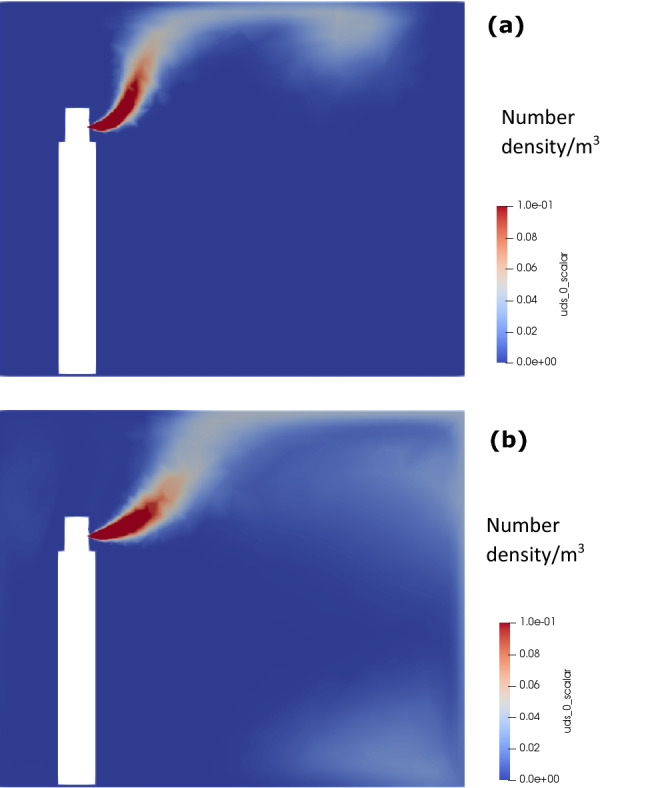
Concentration distribution within the room for continuous talking: **a** 25 s, and **b** 5 min

Figure 9 shows the talking generated aerosol cloud within the room. In this case, a continuous aerosol generation with the combination of natural convection, leads to an upward movement of aerosols cloud, propagating along the roof in the direction of talking jet, eventually striking the other end of the room wall and gradually distributing within the room.

**Fig. 10:**
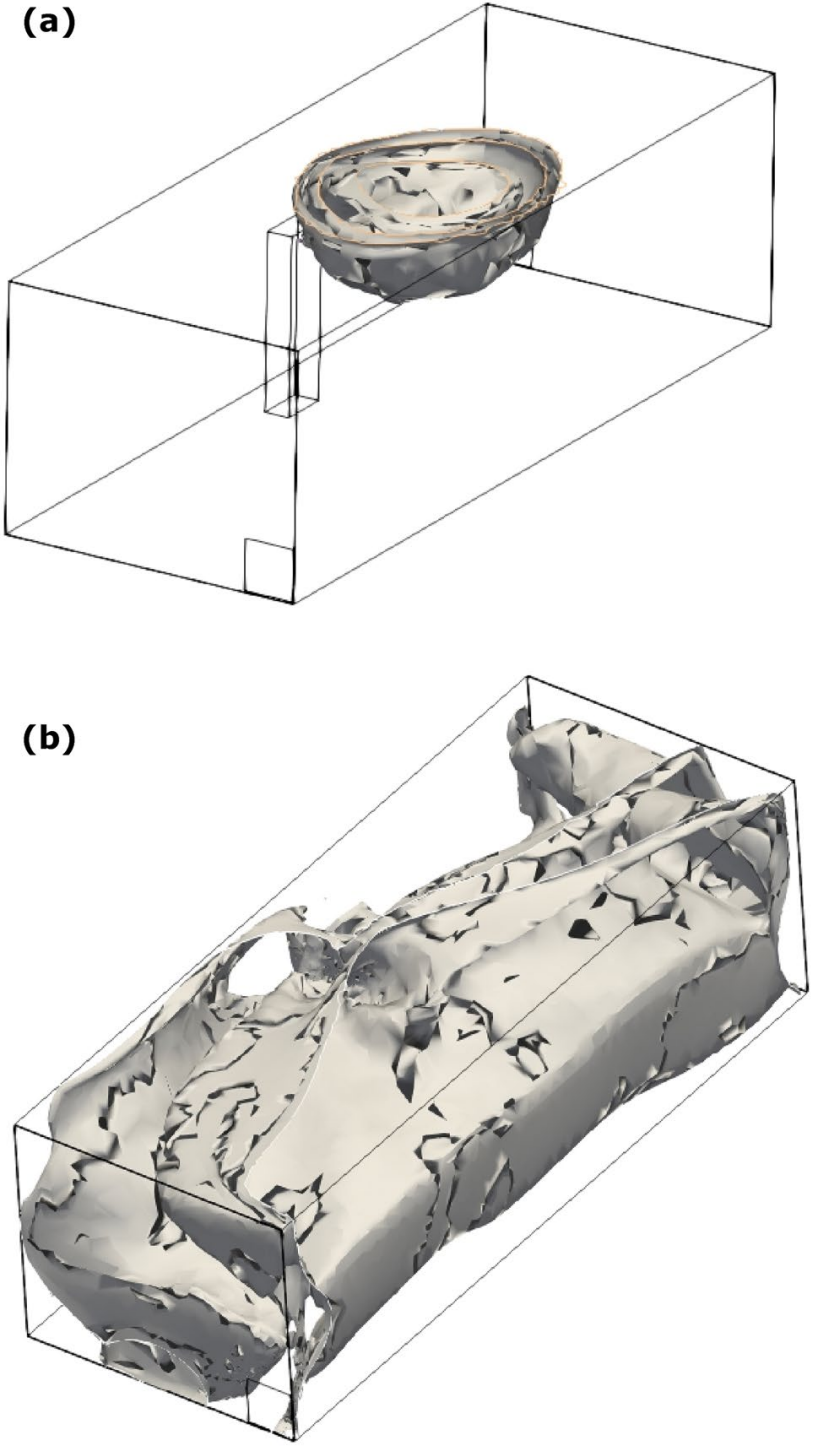
3D contours of concentration for continuous talking: **a** 25 s, and **b** 5 min

Figure 10 shows a wall mounted hemisphere of aerosol clouds within the room that is distributed within the room with a concentration of 10^–4^ after 25 s. Since, the talking is continuously generating aerosols, the aerosol cloud keeps on increasing in size, until the side walls start affecting this distribution, leading to more complicated dispersion of aerosols. The room aerosol concentration will continue to increase beyond 5 min.

**Fig. 11:**
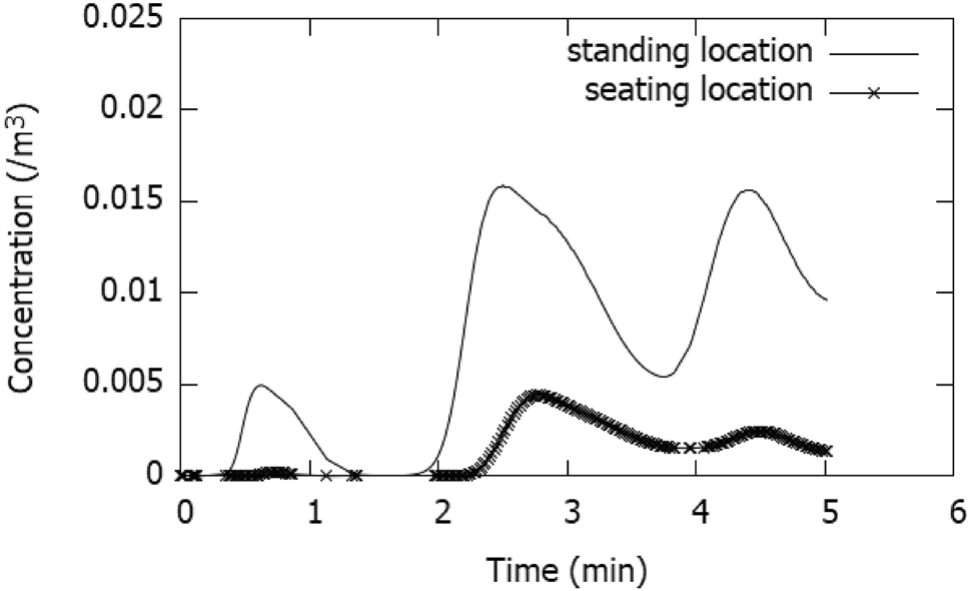
Concentration profile for continuous talking at 2 m from the speaker and 1.6 m (standing location) and 1.05 m (seating location) above the floor

The risk of getting infected if a person is standing and seating at 2 m from the infected person has been investigated using Wells–Riley equation. Figure 11 shows the concentration distribution at two points representing a standing and seating locations which are 2 m in front of the person and at 1.6 m and 1.05 m above the floor. The figure shows the cyclic variation of concentration. This cyclic variation is a result of recirculating flows driven by natural convection. The peak concentration is 0.015 at the standing location, while it is 0.005 at the seating location. The body generated thermal plume creates an upward motion and the aerosol cloud move upwards and then diffuses quickly due to turbulence of natural convection.

**Fig. 12:**
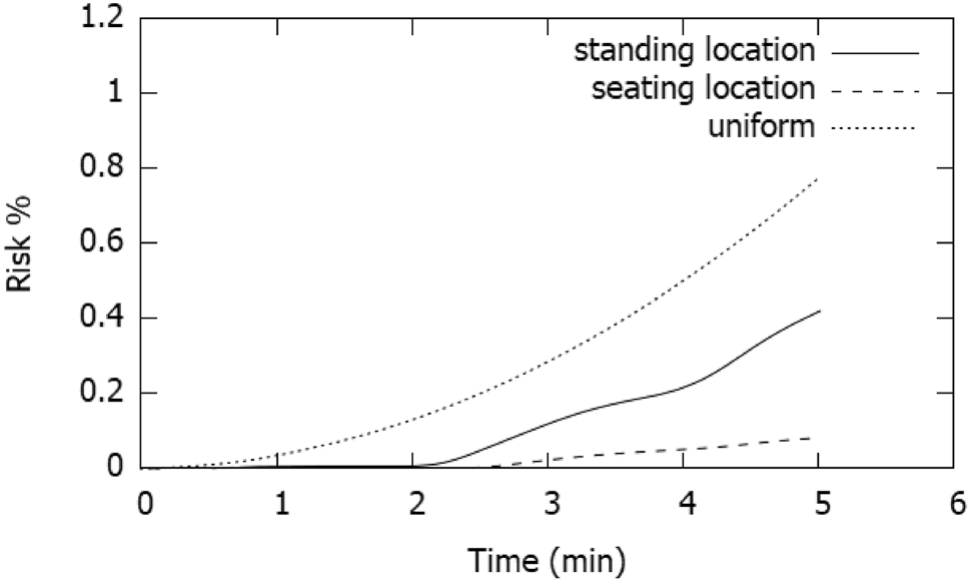
Risk for 5 min exposure of continuous talking at 2 m from the speaker and 1.6 m (standing location) and 1.05 m (seating location) above the floor

Figure 12 shows the risk % at standing and seating locations; the figure shows that after 5 min, at standing locations the risk is 0.4% and at the seating locations the risk is 0.06%. However, the trend of the curves is exponentially upward and thus, the curves can be extrapolated to determine the time required to reach substantial risks. The figure also shows the original Wells–Riley risk profile. The original model is based on uniform distribution of quanta within the room and thus shows a higher risk calculation. The combination of CFD modelling with Wells–Riley equation gives a more nuanced space and time-based risk calculation.

The presented results are based on the simplified model of aerosols transport using the inert scalar concept. The simulation can be made more realistic by tracking the transport of individual droplets within a cloud of droplets and the heat mas transfer model taking into account the transient evaporation model as recently developed by Dbouk and Drikakis (2020b).

## Conclusions

A CFD modelling study has been carried out to investigate the effects of body generated thermal plume on coughing and talking generated aerosol distribution within a room. An inert scalar transport equation has been used to represent aerosol cloud, while turbulence has been modelled with the $k-\epsilon$ turbulence model. A modified Wells–Riley equation has been used to calculate risk of getting infection based on quanta emission concept. From simulation results the following conclusions can be made:

- The present simulation shows that the thermal body generated plume creates a complex recirculatory motion within the room and as a result the coughing and talking generated aerosol cloud moves upwards and its concentration reduces quickly.
- The cough generated aerosols cloud though generated at a higher speed with more aerosol droplets, but occupies a smaller volume within the room after 5 min, while continuous talking generated aerosol cloud dissipates within the whole room within 5 min.
- The risk calculation based on the Wells–Riley model shows a relatively low risk at 2 m distance at standing and seating locations within 5 min, but the nature of the risk profile is exponential upward and thus prolonged exposure will increase the risk substantially.

## Data Availability

The data that support the findings of this study are available from the corresponding author upon reasonable request.
